# Structural and Mechanical Characterization of Zr_58.5_Ti_8.2_Cu_14.2_Ni_11.4_Al_7.7_ Bulk Metallic Glass

**DOI:** 10.3390/ma5010001

**Published:** 2011-12-22

**Authors:** Konda G. Prashanth, Sergio Scudino, Mohsen Samadi Khoshkhoo, Kumar B. Surreddi, Mihai Stoica, Gavin Vaughan, Jürgen Eckert

**Affiliations:** 1 IFW Dresden, Institut für Komplexe Materialien, Postfach 270116, Dresden D-01171, Germany; E-Mails: s.scudino@ifw-dresden.de (S.S.); m.samadi.khoshkhoo@ifw-dresden.de (M.S.K.); surreddi@chalmers.se (K.B.S.); m.stoica@ifw-dresden.de (M.S.); j.eckert@ifw-dresden.de (J.E.); 2 European Synchrotron Radiation Facilities (ESRF), BP 220, Grenoble 38043, France; E-Mail: vaughan@esrf.fr; 3 TU Dresden, Institut für Werkstoffwissenschaft, Dresden D-01062, Germany

**Keywords:** bulk metallic glass (BMG), crystallization, mechanical properties

## Abstract

Thermal stability, structure and mechanical properties of the multi-component Zr_58.5_Ti_8.2_Cu_14.2_Ni_11.4_Al_7.7_ bulk metallic glass have been studied in detail. The glassy material displays good thermal stability against crystallization and a fairly large supercooled liquid region of 52 K. During heating, the alloy transforms into a metastable icosahedral quasicrystalline phase in the first stage of crystallization. At high temperatures, the quasicrystalline phase undergoes a transformation to form tetragonal and cubic NiZr_2_-type phases. Room-temperature compression tests of the as-cast sample show good mechanical properties, namely, high compressive strength of about 1,630 MPa and fracture strain of 3.3%. This is combined with a density of 6.32 g/cm^3^ and values of Poisson’s ratio and Young’s modulus of 0.377 and 77 GPa, respectively. The mechanical properties of the glass can be further improved by cold rolling. The compressive strength rises to 1,780 MPa and the fracture strain increases to 8.3% for the material cold-rolled to a diameter reduction of 10%.

## 1. Introduction

Multi-component bulk metallic glasses (BMGs) are of particular interest for engineering applications because of their positive combination of remarkable mechanical, physical and chemical properties [1,2,3,4,5]. In addition, multi-component BMGs have high thermal stability against crystallization and a wide super cooled liquid region [1,2,3], which helps in the thermoplastic processing of these alloys without crystallization [6].

The complete characterization of BMGs is a necessary prerequisite for their technological application. Metallic glasses are metastable phases and, when heated to a sufficiently high temperature, they tend to a more stable condition, *i.e*., they crystallize [7,8]. Therefore, investigations of the thermal stability of BMGs and their structure evolution during heating is of primary importance not only in order to analyze their stability against crystallization but also for controlling their microstructure, and finally for improving their properties [9]. This is particularly important when partially crystallized materials are considered, where a fundamental parameter that has to be taken into account is the temperature range of stability of the phase formed [10,11].

Another important point of interest in the development of BMGs as structural materials is the evaluation of their mechanical behavior. Metallic glasses show large values of yield stress and elastic strain [1,2,3,5], however, the plastic deformation at room temperature of these materials is generally inhomogeneous and occurs via highly localized shear bands, which finally leads to catastrophic failure with limited microscopic deformability [12]. Catastrophic shear banding in BMGs can be avoided by the creation of heterogeneous microstructures through compositional design to form BMG-matrix composites [13,14] or by the use of the proper mechanical pre-treatments, such as shot peening [15], cold rolling [16,17,18], channel-die compression [19] or elastostatic pre-loading [20]. Besides, for the improvement of the plastic deformability of BMGs, the analysis of the effects of shaping processes, such as rolling or forging, on structure, thermal stability and mechanical properties is of particular interest for the possible implementation of BMGs into a conventional industrial processing line.

In this work, structure and mechanical behavior of the Zr_58.5_Ti_8.2_Cu_14.2_Ni_11.4_Al_7.7_ bulk metallic glass produced by copper mold casting have been investigated in detail. The Zr_58.5_Ti_8.2_Cu_14.2_Ni_11.4_Al_7.7_ metallic glass was selected for the present investigation because this glassy material can be produced by different processing routes [21] and, therefore, it may offer interesting opportunities for a possible commercial application. The thermal stability of the glass was studied by differential scanning calorimetry and the temperature dependence of the structure and the temperature ranges of stability of the different phases formed during heating were investigated by in-situ X-ray diffraction using a high-energy monochromatic synchrotron beam. The mechanical properties of the glass were investigated by room temperature compression tests. Finally, the effectiveness of cold rolling as a mechanical pre-treatment for improving the mechanical behavior and as a suitable shaping process for the present BMG was evaluated.

## 2. Results and Discussion

**Figure 1:**
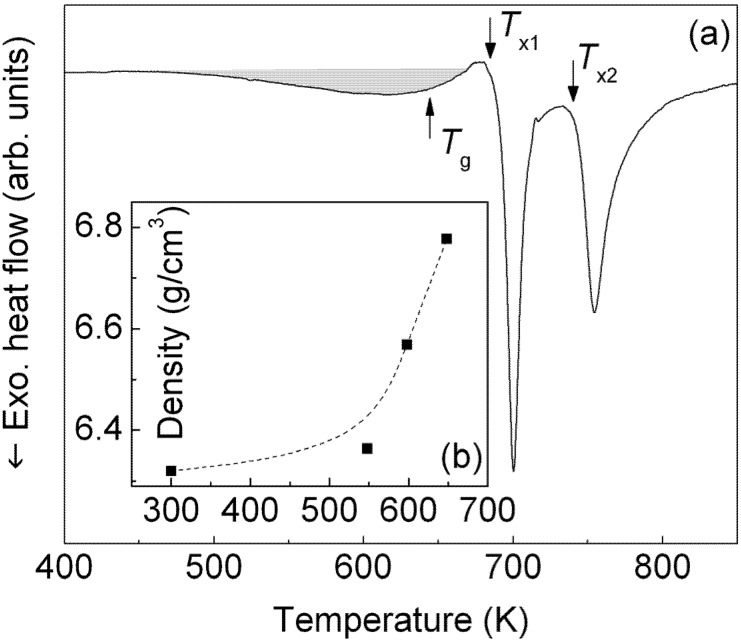
(**a**) Isochronal (40 K/min) DSC scan of the as-cast rod; and (**b**) density of the metallic glass as a function of the annealing temperature.

Figure 1a shows the isochronal (40 K/min) DSC scan of the as-cast Zr_58.5_Ti_8.2_Cu_14.2_Ni_11.4_Al_7.7_ rod. Within the range of temperatures considered in this work, the DSC curve consists of a broad exothermic signal in the range 500–600 K (see shadowed area in Figure 1a), which can be ascribed to structural relaxation [22]. The broad exotherm is followed by an endothermic event associated with the glass transition at *T_g_* = 629 K, which indicates the transformation from the solid-state glass into the supercooled liquid (SCL), before two exothermic heat flow events due to the crystallization of the SCL occur at higher temperatures (*T_x1_* = 681 K and *T_x2_* = 740 K). The supercooled liquid region, *ΔT_x_* = *T_x1_* − *T_g_*, which reflects the thermal stability against crystallization of the SCL, is 52 K. The thermal stability data are in good agreement with those reported in previous studies for melt-spun ribbons and ball-milled powders with the same composition [21] (see Table 1).

**Table 1:** Temperature of the glass transition (*T_g_*), onset of the first (*T_x1_*) and the second (*T_x2_*) crystallization peak and extension of the supercooled liquid region (*ΔT_x_* = *T_x1_* − *T_g_*) for the Zr_58.5_Ti_8.2_Cu_14.2_Ni_11.4_Al_7.7_ cast rod, melt-spun ribbon and ball-milled powder.

	*T_g_* (K)	*T_x1_* (K)	*T_x2_* (K)	*T_x_* (K)	Reference
Cast rod	629	681	740	52	This work
Melt-spun ribbon	637	688	742	51	[21]
Ball-milled powder	636	685	737	49	[21]

**Figure 2:**
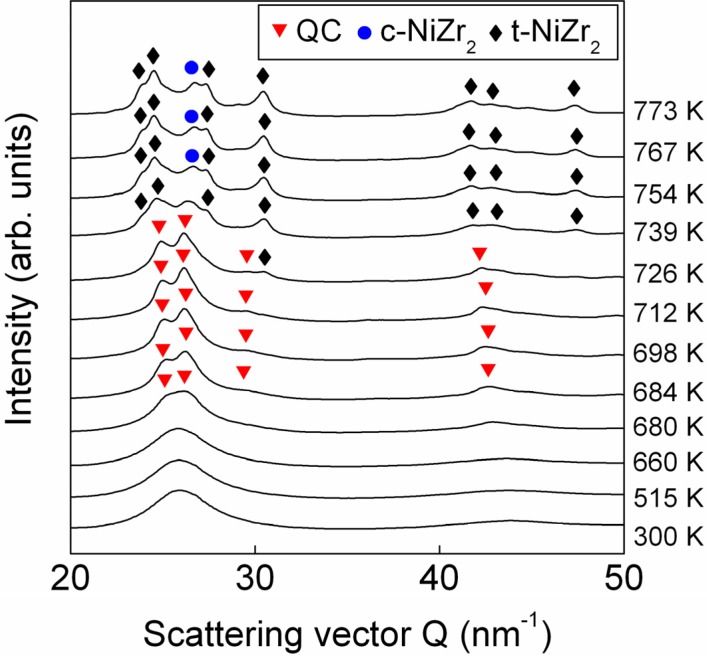
XRD patterns (λ = 0.01249 nm) of the as-cast Zr_58.5_Ti_8.2_Cu_14.2_Ni_11.4_Al_7.7_ bulk metallic glass as a function of temperature.

**Figure 3:**
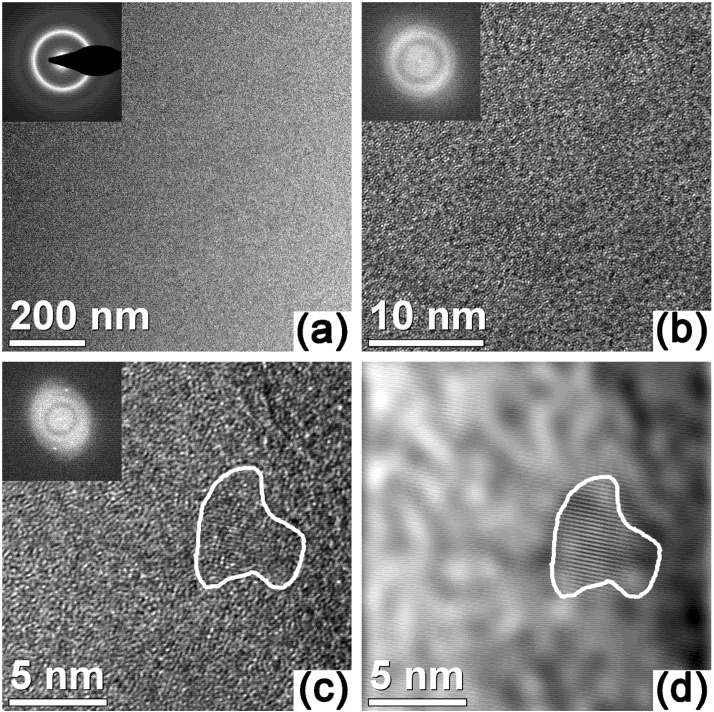
TEM data for the as-cast Zr_58.5_Ti_8.2_Cu_14.2_Ni_11.4_Al_7.7_ glassy alloy: (**a**) bright-field image of the as-cast material and (**inset**) corresponding selected area electron diffraction pattern; (**b**,**c**) high-resolution TEM micrographs and (**insets**) corresponding fast Fourier transforms (FFT); (**d**) inverse FFT of Figure 3(c) after the Fourier mask filtering.

The XRD pattern of the as-cast rod taken at room temperature is shown in Figure 2 (300 K). The pattern displays only the broad diffuse maxima typical for an amorphous material without additional peaks due to crystalline phases. The amorphous nature of the as-cast material is confirmed by the TEM results in Figure 3a. The bright-field image is featureless with no traces of diffraction contrast due to second-phase particles. In addition, the corresponding selected area electron diffraction pattern (inset in Figure 3a) shows the typical diffuse diffraction rings characteristic of amorphous materials. As well, high-resolution TEM (Figure 3b) shows the characteristic disordered structure of amorphous materials and no bright spots indicative of the presence of crystalline phases can be observed in the fast Fourier transform (FFT) of the image (inset in Figure 3b). Only few and dispersed ordered regions of about 3–5 nm can be observed at a higher magnification (Figure 3c). This is particularly clear in Figure 3d, which shows the inverse FFT of Figure 3c after the Fourier mask filtering.

The structure evolution of the Zr_58.5_Ti_8.2_Cu_14.2_Ni_11.4_Al_7.7_ bulk metallic glass during heating is shown in Figure 2, where the sequence of individual scans collected at different temperatures illustrating diffracted intensity vs. scattering vector (*Q* = 4πsinθ/λ) are plotted as a function of temperature. The XRD patterns reveal that, besides the amorphous phase, no additional phases are formed up to 660 K. When the sample is heated to 680 K, corresponding to the onset of the first crystallization DSC peak in Figure 1a, the pattern displays the presence of additional diffraction contributions at *Q* = 25, 26 and 43 nm^−1^ that overlap to the main amorphous maxima. The occurrence of these small, broad diffraction peaks indicates the formation of an ordered phase of nanoscale dimensions. The intensity of the small peaks grows with increasing temperature, which permits to unambiguously identify the phase formed at temperatures above 680 K as an icosahedral quasicrystalline (QC) phase. In addition to the diffraction signals of the QC phase, the diffuse diffraction maximum of the glassy phase is visible, indicating the presence of a residual amorphous phase. The QC phase is metastable and transforms to NiZr_2_-type phases with cubic (space group $Fd\bar{3}m$ ) and tetragonal (space group I4/mcm) crystalline structures at temperatures above 726 K (coinciding with the second crystallization event). A similar structural evolution has been observed for the corresponding alloy with the same composition produced by melt spinning and ball milling [21].

**Figure 4:**
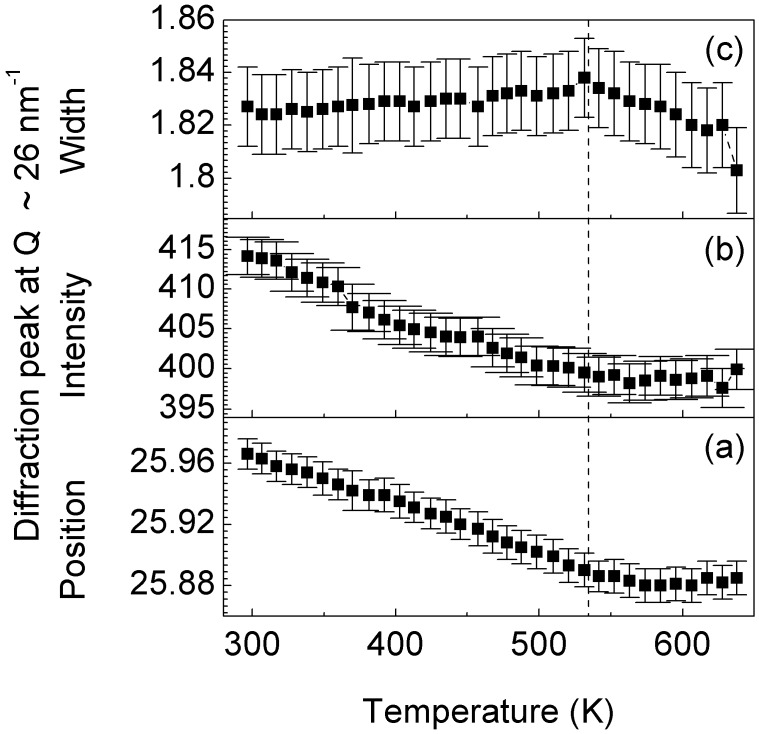
Temperature dependence of the (**a**) position; (**b**) intensity; and (**c**) width of the main amorphous maximum at about *Q* = 26 nm^−1^ in Figure 2.

Although no phase changes can be observed in Figure 2 at temperatures below 680 K, the glassy structure undergoes structural changes in this temperature regime. This behavior can be observed in Figure 4, which shows the temperature dependence of the position, intensity and width of the main amorphous maximum at about *Q* = 26 nm^−1^. The position of the amorphous maximum, decreases linearly with increasing temperature up to about 530 K (Figure 4a) as a result of the thermal expansion, which is linked to the increase of the mean atomic spacing (*i.e*., dilatation) [23]. Above 530 K, at temperatures corresponding to the broad exothermic event in Figure 1a, the peak position remains almost constant. This behavior can be attributed to the additional effect of free volume annihilation that, by inducing shorter atomic distances and thus densification [23], counterbalances the effect of thermal expansion. An analogous effect can be observed for the intensity and width of the amorphous maximum (Figure 4b and c). At temperatures below 530 K, the intensity decreases and the width increases with increasing temperature. This is due to the Debye-Waller effect, which describes the attenuation of the X-ray scattering caused by the thermal agitation [24]. Above 530 K, this effect is no longer visible due to the additional contribution of the free volume annihilation and to the consequent densification of the material, as demonstrated by the increase of the density of the metallic glass with increasing annealing temperature (Figure 1b).

**Figure 5:**
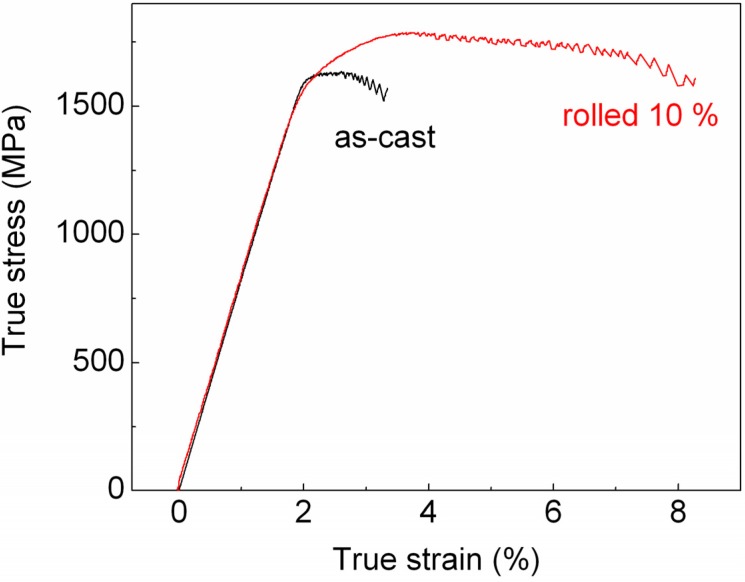
Room temperature stress-strain curves for the as-cast and cold-rolled Zr_58.5_Ti_8.2_Cu_14.2_Ni_11.4_Al_7.7_ BMG under quasistatic compressive loading.

A typical room temperature stress-strain curve for the as-cast Zr_58.5_Ti_8.2_Cu_14.2_Ni_11.4_Al_7.7_ BMG under quasistatic compressive loading is shown in Figure 5. The specimen exhibits an elastic regime of 1.85% before yielding, which occurs at *σ*_y_~1,500 MPa. The Young’s modulus, estimated by ultrasonic measurements, is 77 GPa. After yielding the stress increases with increasing strain and the sample shows an apparent work-hardening behavior up to 1,630 MPa and 2.6% strain. With further increase of strain, the stress-strain curve displays a work-softening behavior up to fracture, which occurs at *σ*_f_ = 1,570 MPa and at *ε*_f_ = 3.3% strain.

The occurrence of large Poisson’s ratios has been proposed for explaining the enhanced plastic deformability of some BMGs [25,26]. For materials with a large Poisson’s ratio, the release of local stress concentrations is more likely to occur via shear deformation rather than through crack nucleation, leading to improved plastic deformation [5]. More specifically, metallic glasses having values of Poisson’s ratio larger than 0.31–0.32 are tough, while BMGs with smaller values are brittle [26]. The present Zr_58.5_Ti_8.2_Cu_14.2_Ni_11.4_Al_7.7_ BMG displays a Poisson’s ratio of 0.377 and, therefore, it falls into the category of the tough BMGs.

The room-temperature plasticity observed in Figure 5 may also be linked to the presence of the nanocrystals in the as-cast structure (Figure 3c and d). In certain BMGs derived from shape memory alloys, a deformation-induced precipitation of nanocrystals has been suggested as a process competing with the formation of the shear transformation zones (the fundamental units of plasticity in metallic glasses at low temperatures [27]), which leads to a small plastic strain even in tension [28]. This mechanism may be active during deformation of the Zr_58.5_Ti_8.2_Cu_14.2_Ni_11.4_Al_7.7_ BMG. Although few in number, the nanocrystals may nevertheless interfere with the process of irreversible deformation, effectively limiting shear bands from propagating catastrophically and explaining the observed macroscopic plastic deformation.

**Figure 6:**
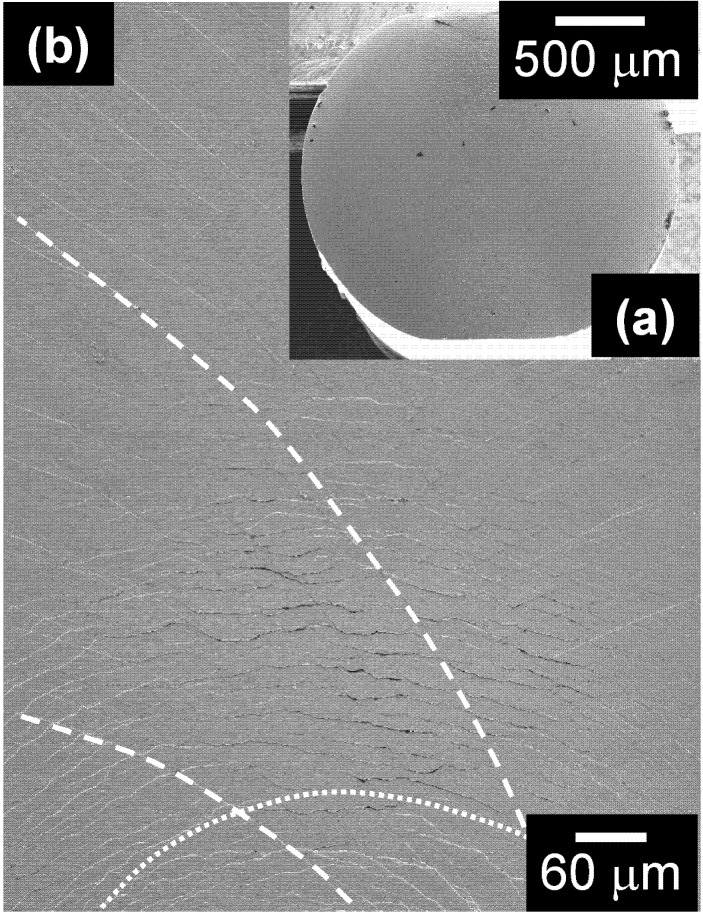
Cross-section of the metallic glass cold-rolled to a diameter reduction of 10%, revealing the formation of different types of shear bands.

The Zr_58.5_Ti_8.2_Cu_14.2_Ni_11.4_Al_7.7_ metallic glass not only displays interesting mechanical properties under compressive loading, but it also shows good damage tolerance when subjected to cold rolling. The cylindrical rod can be cold-rolled up to a diameter reduction of 10% without inducing visible cracks and, as a result, the cross-section of the sample is no longer round, as for the as-cast sample, but it displays two flat surfaces of about 600–700 μm (Figure 6a). Plastic deformation during rolling is accommodated by the formation of two distinct families of shear bands (Figure 6b): semicircular shear bands originating from the flat surfaces (indicated by dotted lines in Figure 6b) and curved shear bands, which intersect the semicircular shear bands and extend towards the centre of the sample (dashed lines in Figure 6b). Such a shear band morphology is in agreement with that observed for other cold-rolled Zr-based cylindrical BMGs [17,18] and can be ascribed to the heterogeneous plastic deformation that characterizes rolling of cylindrical specimens.

Besides for cold working and shaping of materials, cold rolling can be used to improve the mechanical behavior of BMGs [16,18]. The mechanical properties of the present Zr_58.5_Ti_8.2_Cu_14.2_Ni_11.4_Al_7.7_ BMG are indeed improved by cold rolling (Figure 5). The yield strength of the material cold-rolled up to a diameter reduction of 10% is about 1,470 MPa, only slightly reduced with respect to the as cast material (1,500 MPa). On the other hand, the compressive strength rises from 1,630 MPa for the as-cast material to 1,780 MPa for the cold-rolled sample, which gives rise to a clear work-hardening behavior. As well, rolling has a significant effect on the fracture strain, which increases from 3.3% for the as-cast sample to 8.3% for the rolled material. A possible explanation for the improved mechanical behavior is given by the propagation-arrest mechanism of shear bands resulting from the creation of a heterogeneous microstructure consisting of hard and soft regions during rolling [18]. Most likely, the rolling-induced hard and soft regions necessitate different critical stresses to initiate deformation during the subsequent compressive test. The deformation of the soft regions may be activated at low stress, explaining the slightly lower yield strength of the cold-rolled sample compared to the as-cast material [12,18]. On the other hand, the hard regions may hinder or stop the propagation of the shear bands previously formed in the soft areas [12,18]. In order to accommodate further strain, new shear bands have to be generated in the hard regions, which require increasingly higher critical stresses for shear band nucleation and propagation [18], explaining the work-hardening behavior visible in Figure 5. This propagation-arrest mechanism may assist initiation, branching and arresting of multiple shear bands effectively impeding a single shear band from propagating catastrophically [17,18] and resulting in the improved plastic deformation characterizing the cold-rolled material with respect to the as-cast samples.

## 3. Experimental Section

An ingot with nominal composition Zr_58.5_Ti_8.2_Cu_14.2_Ni_11.4_Al_7.7_ (purity > 99.9 wt %) was prepared by arc melting in a titanium-gettered argon atmosphere. The ingot was remelted several times in order to achieve a homogeneous master alloy. From this ingot, cylindrical bulk samples with 2 mm diameter and 80 mm length were prepared by copper mold casting. The density of the samples was evaluated by the Archimedes principle. The thermal stability of the samples was investigated by differential scanning calorimetry (DSC) with a Perkin-Elmer DSC7 calorimeter at 40 K/min heating rate under a continuous flow of purified argon. The calorimeter was calibrated for temperature and enthalpy with high purity Indium and Zinc, giving an experimental error for the temperature and enthalpy of less than 1 K and 0.5 J/g, respectively. The structure evolution of the as-cast rod during heating was studied by x-ray diffraction (XRD) in transmission configuration using a high-energy monochromatic synchrotron beam (λ = 0.01249 nm) at the ID11 beamline of the European Synchrotron Radiation Facilities (ESRF). Diffraction data were collected at a constant heating rate of 40 K/min in order to compare the structural evolution with the thermal stability investigated by DSC. The structure of the as-cast sample was also investigated by using transmission electron microscope (TEM) using a Tecnai F30 transmission electron microscope operating at 300 kV. For the preparation of the TEM specimens, a thin slice of the as-cast rod was thinned down to 100 µm by grinding. It was then dimple grinded using a Gatan dimple grinding machine. Ion milling (Gatan-PIPS ion miller) was used to remove all mechanical damages and distortions introduced by the previous steps. Cylindrical specimens of 2 mm diameter and 4 mm length were prepared from the as-cast rod and tested with an Instron 8562 testing facility under quasistatic loading (strain rate ~1 × 10^−4^ s^−1^) at room temperature. Both ends of the specimens were polished to make them parallel to each other prior to the compression test. The strain during the compression tests was measured directly on the specimen using a Fiedler laser-extensometer. The as-cast cylindrical samples were cold-rolled at room temperature to a diameter reduction of 10% using a laboratory rolling mill. The surface morphology of the specimens was evaluated by scanning electron microscopy (SEM) using a Gemini 1530 microscope. Poisson’s ratio and Young’s modulus were evaluated by ultrasonic measurements using an Olympus 5900 PR ultrasonic pulser-receiver.

## 4. Conclusions

Thermal stability, structure evolution during heating and mechanical properties of the Zr_58.5_Ti_8.2_Cu_14.2_Ni_11.4_Al_7.7_ bulk metallic glass prepared by copper mold casting, have been extensively investigated. The thermal stability of the glass was studied by differential scanning calorimetry, and the temperature dependence of the structure was investigated by *in-situ* X-ray diffraction using a high-energy monochromatic synchrotron beam. The results reveal that the crystallization behavior of the present metallic is characterized by the annihilation of the free volume at temperatures below the glass transition resulting in the densification of the material. The glass displays good thermal stability against crystallization and a fairly large supercooled liquid region of 52 K followed by a double-step devitrification behavior characterized by the precipitation of a metastable quasicrystalline phase in the first stage of the crystallization process and by the formation of tetragonal and cubic NiZr_2_-type crystalline phases in the following crystallization event. Room temperature compression tests of the as-cast sample show good mechanical properties, namely, high compressive strength of about 1,630 MPa and fracture strain of 3.3%. This is combined with a density of 6.32 g/cm^3^ and values of Poisson’s ratio and Young’s modulus of 0.377 and 77 GPa, respectively. The as-cast glass can be cold-rolled up to a dimensional change of about 10% without macroscopic damage. The cold-rolled material displays improved mechanical properties compared with the as-cast metallic glass, the strength rises to 1,780 MPa and the fracture strain increases to 8.3%. This not only offers the possibility to cold work the present BMG, but it gives a method to further improve the mechanical properties of the material.

## References

[1] Inoue A. (2000). Stabilization of metallic supercooled liquid and bulk amorphous alloys. Acta Mater..

[2] Peker A., Johnson W.L. (1993). A highly processable metallic glass: Zr_41.2_Ti_13.8_Cu_12.5_Ni_10_Be_22.5_. Appl. Phys. Lett..

[3] Johnson W.L. (1999). Bulk glass-forming metallic alloys: Science and technology. MRS Bull..

[4] Gebert A., Mummert K., Eckert J., Schultz L., Inoue A. (1997). Electrochemical investigations on the bulk glass forming Zr_55_Cu_30_Al_10_Ni_5_ alloy. Mater. Corros. 1997, 48,.

[5] Eckert J., Das J., Pauly S., Duhamel C. (2007). Mechanical properties of bulk metallic glasses and composites. J. Mater. Res..

[6] Kumar G., Tang H.X., Schroers J. (2009). Nanomoulding using thermoplastic forming with bulk metallic glass. Nature.

[7] Scott M.G., Luborsky F.E. (1983). Crystallization. Amorphous Metallic Alloys.

[8] Prashanth K.G., Scudino S., Murty B.S., Eckert J. (2009). Crystallization kinetics and consolidation of mechanically alloyed Al_70_Y_16_Ni_10_Co_4_ glassy powders. J. Alloys Comp..

[9] Eckert J., Scudino S., Groza J.R., Schackelford J.F., Lavernia E.J., Powers M.T. (2007). Crystallization of metallic glasses. Materials Processing Handbook.

[10] Scudino S., Venkataraman S., Sakaliyska M., Eckert J. (2008). Crystallization behavior and consolidation of ball milled Zr_60_Ti_5_Ag_5_Cu_12.5_Ni_10_Al_7.5_ glassy powders. J. Alloys Comp..

[11] Scudino S., Eckert J., Mickel C., Schubert-Bischoff P., Breitzke H., Lüders K., Schultz L. (2005). Quasicrystalline phase formation in Zr-Ti-Nb-Cu-Ni-(Al) metallic glasses. J. Alloys Comp..

[12] Chen M. (2008). Mechanical behavior of metallic glasses: Microscopic understanding of strength and ductility. Annu. Rev. Mater. Res..

[13] Hofmann D.C., Suh J.-Y., Wiest A., Duan G., Lind M.-L., Demetriou M.D., Johnson W.L. (2008). Designing metallic glass matrix composites with high toughness and tensile ductility. Nature.

[14] Wu Y., Xiao Y., Chen G., Liu C.T., Lu Z. (2010). Bulk metallic glass composites with transformation-mediated work-hardening and ductility. Adv. Mater..

[15] Zhang Y., Wang W.H., Greer A.L. (2006). Making metallic glasses plastic by control of residual stress. Nat. Mater..

[16] Lee M.H., Lee K.S., Das J., Thomas J., Kühn U., Eckert J. (2010). Improved plasticity of bulk metallic glasses upon cold rolling. Scripta Materialia.

[17] Scudino S., Surreddi K.B., Eckert J. (2010). Mechanical properties of cold-rolled Zr_60_Ti_5_Ag_5_Cu_12.5_Ni_10_Al_7.5_ metallic glass. Phys. Stat. Sol..

[18] Scudino S., Jerliu B., Surreddi K.B., Kühn U., Eckert J. (2011). Effect of cold rolling on compressive and tensile mechanical properties of Zr_52.5_Ti_5_Cu_18_Ni_14.5_Al_10_ bulk metallic glass. J. Alloys Comp..

[19] Scudino S., Surreddi K.B., Samadi Khoshkhoo M., Sakaliyska M., Wang G., Eckert J. (2010). Improved room temperature plasticity of Zr_41.2_Ti_13.8_Cu_12.5_Ni_10_Be_22.5_ bulk metallic glass by channel-die compression. Adv. Eng. Mater..

[20] Lee S.C., Lee C.M., Yang J.W., Lee J.C. (2008). Microstructural evolution of an elastostatically compressed amorphous alloy and its influence on the mechanical properties. Scripta Materialia.

[21] Scudino S., Eckert J., Mickel C., Schultz L. (2005). On the amorphous-to-quasicrystalline phase transformation in ball-milled and melt-spun Zr_58.5_Ti_8.2_Cu_14.2_Ni_11.4_Al_7.7_ glassy alloys. J. Non-Cryst. Solids.

[22] Venkataraman S., Scudino S., Eckert J., Gemming T., Mickel C., Schultz L., Sordelet D.J. (2006). Nanocrystallization of gas atomized Cu_47_Ti_33_Zr_11_Ni_8_Si_1_ metallic glass. J. Mater. Res..

[23] Yavari A.R., le Moulec A., Inoue A., Nishiyama N., Lupu N., Matsubara E., Botta W.J., Vaughan G., di Michiel M., Kvick Å. (2005). Excess free volume in metallic glasses measured by X-ray diffraction. Acta Mater..

[24] Guinier A. (1994). X-Ray Diffraction in Crystals, Imperfect Crystals and Amorphous Bodies.

[25] Schroers J., Johnson W.L. (2004). Ductile bulk metallic glass. Phys. Rev. Lett..

[26] Lewandowski J.J., Wang W.H., Greer A.L. (2005). Intrinsic plasticity or brittleness of metallic glasses. Phil. Mag. Lett..

[27] Argon A.S. (1979). Plastic deformation in metallic glasses. Acta Metall..

[28] Pauly S., Gorantla S., Wang G., Kühn U., Eckert J. (2010). Transformation-mediated ductility in CuZr-based bulk metallic glasses. Nat. Mater..

